# Relationship between autoimmune thyroid antibodies and anti-nuclear antibodies in general patients

**DOI:** 10.3389/fendo.2024.1368088

**Published:** 2024-03-25

**Authors:** Yi Ruan, Xian-pei Heng, Liu-qing Yang, Wei-dong He, Liang Li, Zhi-ta Wang, Su-ping Huang, Qi-wei Chen, Zhuang Han

**Affiliations:** ^1^ Department of Endocrinology, People’s Hospital Affiliated to Fujian University of Traditional Chinese Medicine, Fuzhou, China; ^2^ First Clinical Medical College , Fujian University of Traditional Chinese Medicine, Fuzhou, China; ^3^ Department of Geriatrics, People’s Hospital Affiliated to Fujian University of Traditional Chinese Medicine, Fuzhou, China; ^4^ Journal Office of Fujian University of Traditional Chinese Medicine, Fuzhou, China

**Keywords:** Graves’ disease, Hashimoto thyroiditis, autoimmune thyroid antibodies, anti-nuclear antibodies, NHANES

## Abstract

**Background:**

There is no doubt that both Hashimoto thyroiditis and Graves’ disease are autoimmune thyroid diseases (AITDs), but the relationship between anti-nuclear antibody (ANA) and AITDs is poorly studied. The association between thyroid autoantibody levels and ANA positivity was evaluated to assess the role of ANA in AITDs.

**Methods:**

We conducted an analysis using data from 1,149,893 patients registered at our hospital and 53,021 patients registered in the National Health and Nutrition Examination Survey databases. We focused on patients with data for thyroid peroxidase antibody (TPOAb)/ANA, TPOAb/immunoglobulin G (IgG), thyroid-stimulating hormone (TSH) receptor antibody (TRAb)/ANA, TRAb/IgG, TSH/ANA, or TSH/IgG.

**Results:**

ANA positivity rates were 12.88% and 21.22% in TPOAb/ANA and TSH/ANA patients, respectively. In TPOAb/IgG and TSH/IgG data, high IgG levels (≥15 g/L) were detected in 2.23% and 4.06% of patients, respectively. There were significant differences in ANA positivity rates and high IgG proportions among patients with different TPOAb and TSH levels. TPOAb level was correlated with ANA positivity rate and high IgG proportion, and TSH level was correlated with ANA positivity rate. Regression analysis showed positive correlations between TPOAb levels and ANA positivity risk or high IgG risk, TSH levels and high IgG risk, and elevated TSH and ANA positivity risk. Of patients with TRAb/ANA data, 35.99% were ANA-positive, and 13.93% had TRAb levels ≥1.75IU/L; 18.96% of patients with TRAb/IgG data had high IgG levels, and 16.51% had TRAb levels ≥1.75IU/L. ANA positivity rate and high IgG proportion were not significantly different among different TRAb levels. TRAb levels, ANA positivity risk and high IgG risk were not correlated.

**Conclusion:**

ANA positivity and high IgG are related to Hashimoto thyroiditis but not Graves’ disease, which implies distinct pathophysiological mechanisms underlying the AITDs.

## Introduction

Anti-nuclear antibodies (ANAs) are non-organic nuclear antibodies produced during the humoral immune response. In 2019, the European League Against Rheumatism/American College of Rheumatology endorsed “positive ANA (titer ≥ 1:80 by HEp-2 immunofluorescence)” as an entry criterion for systemic lupus erythematosus (SLE) (1). This criterion was suitable for SLE classification in patients from south China (2). Furthermore, patients with SLE exhibit elevated levels of total immunoglobulin G (IgG) and ANA IgG subclasses compared with those with other autoimmune diseases (3). Pan et al. reported increased thyroid autoantibodies in patients with SLE (4), and Lin et al. demonstrated an increased risk of new-onset SLE in individuals with Hashimoto thyroiditis (HT) (5). Patients with HT display seropositivity for thyroid peroxidase antibody (TPOAb), an HT biomarker, and thyroglobulin antibodies due to the presence of thyroid peroxidase (TPO) and thyroglobulin antigens. Notably, HT and SLE share similar genetic features and are associated with the major histocompatibility complex class II (6, 7). Additionally, patients with rheumatoid arthritis have a predisposition to develop hypothyroidism (8). Thyroid-stimulating hormone (TSH) receptor antibody (TRAb)-IgG levels are higher in patients with Graves’ disease (GD) than in healthy controls (9). ANA levels are also significantly elevated in patients with autoimmune thyroid diseases (AITDs) compared with in healthy individuals (10).

Previous findings have demonstrated that thyroglobulin is not located in the nucleus; instead, it is a cell surface antigen involved in complement-mediated cytotoxicity. Similarly, TRAb is found on cell surfaces and not within the nucleus. Thushani et al. reported elevated TPOAb levels in patients with ANA-positive diseases, such as SLE, compared with in healthy individuals (11). Conversely, high ANA levels have been observed in patients with AITDs (12).

In patients with HT, thyroid follicular cells express intercellular adhesion molecule-1(ICAM-1) (13), and T cells bind to these molecules on target cells, a fundamental component of any immune response (14). However, it remains uncertain whether thyroid follicular cells express ICAM-1 in patients with GD. Furthermore, the pathogenesis of AITD is reportedly linked to the responsiveness of T cells to TPO (15). Teng et al. noted a lack of abnormal distribution of T cell receptor genes in thyroid-derived lymphocytes in patients with GD; however, T cell receptor gene rearrangement occurs in the thyroid glands of patients with HT (16). Davis et al. found marked limitations of *Vα* gene expression in GD, albeit few limitations in HT (17). Thus, the immune mechanisms underlying HT and GD differ considerably. However, the association of ANA positivity with AITD markers, such as TPOAb, TRAb, and TSH levels, has not been evaluated in the general patient population. Therefore, our study aimed to investigate the relationship between ANA positivity and various thyroid autoantibody levels to elucidate whether ANAs play a role in the pathological processes of AITD.

## Materials and methods

### Study design

The Ethics Review Committee of the People’s Hospital Affiliated to Fujian University of Traditional Chinese Medicine approved the study (approval number 2023-031-01), which adhered to the principles of the Declaration of Helsinki. The study did not impede the personal privacy of patients and business interests. The requirement for informed consent was waived due to the retrospective design of the study. All research data were collected through the Scientific Research Integration Platform of our hospital. Additionally, de-identified patient data were obtained from the National Health and Nutrition Examination Survey (NHANES) database, including NHANES questionnaires, datasets, and related documentation, spanning NHANES 1999-2004 and 2007-2012 laboratory data, accessible via the NHANES homepage [cdc.gov].

**Figure 1:**
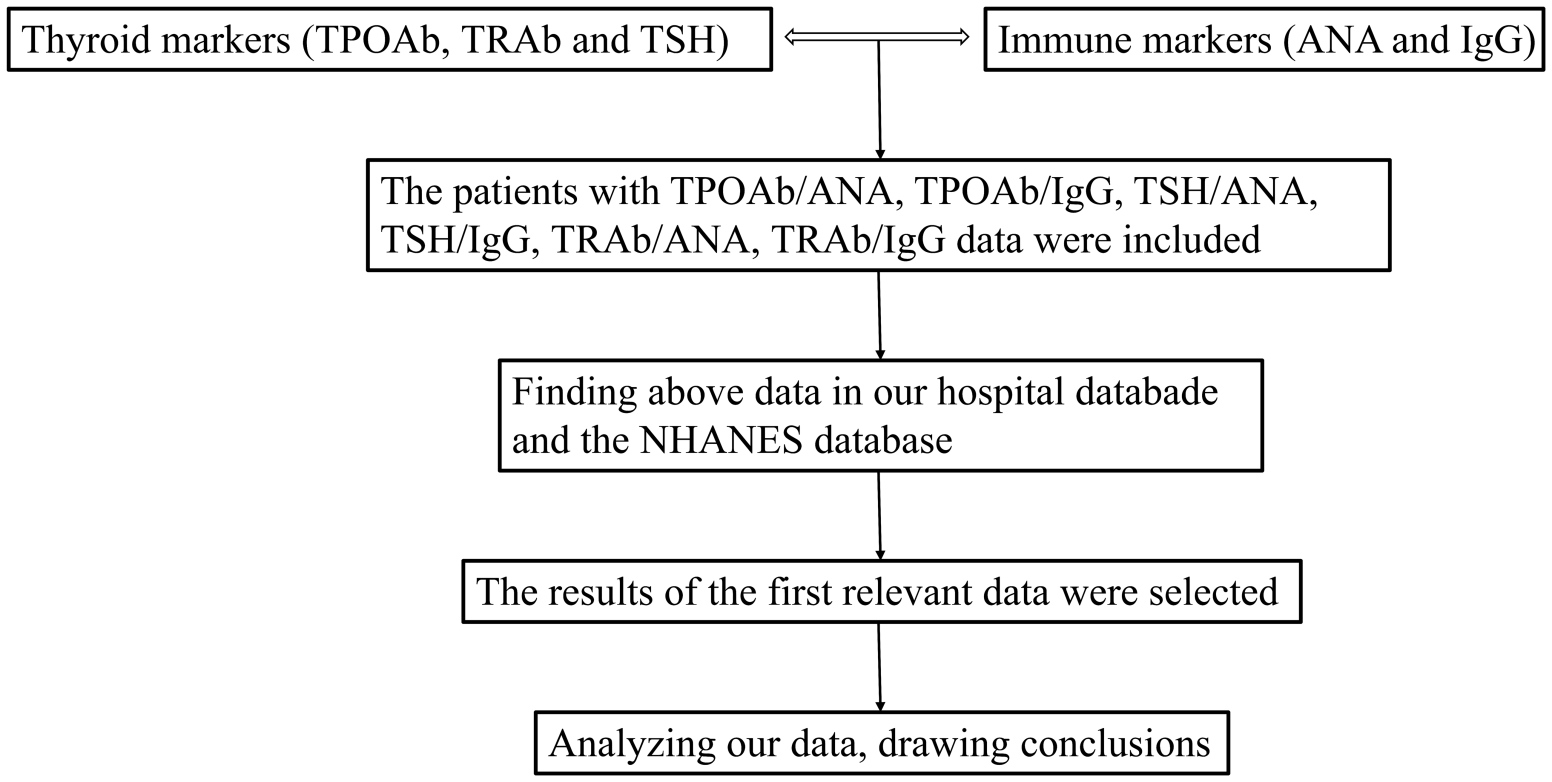
Study flowchart. TPOAb, thyroid peroxidase antibodies; ANA, anti-nuclear antibody; IgG, immunoglobulin G; TSH, thyroid-stimulating hormone; TRAb, thyroid-stimulating hormone receptor antibodies.

Data from the included patients was extracted, organized and analyzed (**Figure 1**).

Inclusion criteria: (1) patients in the Scientific Research Integration Platform of our hospital, and in the NHANES database; (2) TPOAb, TRAb, TSH, ANA, and IgG data tested first time; (3) data from April 1, 2016 to March 31, 2022 in our hospital, and from 1999-2004 and 2007-2012 in the NHANES.

Exclusion criteria: (1) the data of TPOAb, TRAb, TSH, ANA, and IgG retested; (2) the data not within the set time; (3) human induced hypoglobulinemia by controlling food.

### Data sources and study cohort

The Scientific Research Integration Platform of People’s Hospital Affiliated to Fujian University of Traditional Chinese Medicine is specialty software designed for scientific research purposes. This software has been used to collect anonymized patient diagnosis and treatment information since 2003. We included cases from April 1, 2016 to March 31, 2022, in the study. Our study cohort comprised individuals with available data on combinations of TPOAb and ANA, TPOAb and IgG, TRAb and ANA, TRAb and IgG, TSH and ANA, or TSH and IgG levels. For each individual, we selected the results from the first relevant test, Additionally, we included patients with data on ANA, TPOAb, TRAb, TSH, and IgG levels from the NHANES database.

### Thyroid markers, ANA, and IgG detection

Detection of TPOAb, TRAb, and TSH were performed using chemiluminescence immunoassay with the Abbott automatic immunoassay analyzer (Abbott; Chicago, IL, USA). ANA detection was performed using immunofluorescence with the OUMENG reagent (EUROIMMUN Medical Diagnostics., Beijing, China). IgG detection was performed via immunoscattering turbidimetry using the Beckman-specific protein analyzer (Beckman Coulter, Inc.; Brea, CA, USA).

### Outcomes

Our primary outcome involved assessing ANA positivity and negativity rates across various TPOAb, TRAb, and TSH levels. We subsequently calculated the risk of ANA positivity for different TPOAb, TRAb, and TSH levels. As a secondary outcome, we examined the proportion of patients with IgG ≥15 g/L in relation to various TPOAb, TRAb, and TSH levels. We then predicted the risk of having high IgG (≥15 g/L) for different TPOAb, TRAb, and TSH levels.

### Statistical analyses

Statistical analyses were performed using SPSS version 26.0 (IBM Corp.; Armonk, NY, USA). Qualitative variables are presented as percentages and were assessed using the chi-square test. Kendall’s tau-b analysis was also performed for the inter-indicator correlation. Logistic regression analysis was employed to determine the risk factor for ANA positivity and high IgG levels. Fisher’s exact test was used for qualitative variables with an expected value < 5. P < 0.05 was considered statistically significant. Additionally, odds ratio (OR) and corresponding 95% confidence intervals (CIs) were calculated. Graphs were generated using GraphPad Prism 8 (GraphPad Software Inc., San Diego, CA, USA).

In the primary analysis, TPOAb, TRAb, and TSH levels were categorized based on the normal reference ranges provided by the hospital laboratory department and the target levels recommended by the guidelines. TPOAb levels 1, 2, and 3 were defined as < 150, 150 ≤ TPOAb ≤ 600, and TPOAb > 600 IU/mL, respectively. TRAb levels 1 and 2 were defined as < 1.75 and ≥ 1.75 IU/L, respectively. TSH levels 1, 2, and 3 were defined as <0.3, 0.3 ≤ TSH ≤6, and TSH >6 mIU/L, respectively.

## Results

### Study population

Out of the 1,149,893 individuals (529,592 males and 620,301 females) who presented at our hospital, 5396 (2274 males and 3122 females) met the eligibility criteria for inclusion in this study. Additionally, data for 14995 individuals were retrieved from 53,021 laboratory data questionnaires in the NHANES 1994-2004 and 2007-2012 databases. The data obtained from the hospital’s scientific research platform and the NHANES database were found to be comparable. Consequently, a total of 20391 patients with available data on TPOAb and ANA, TPOAb and IgG, TSH and IgG, or TSH and ANA were included in the study.

### TPOAb and ANA

Data on TPOAb and ANA were accessible for 13,946 patients (12,502 from the NHANES database), resulting in an ANA positivity rate of 12.88% (1796/13,946).

**Figure 2:**
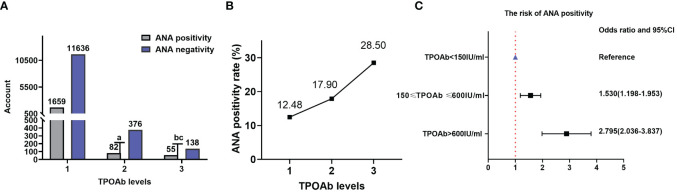
ANA and TPOAb levels. **(A)** Comparison of the ANA positivity and negativity rates at different TPOAb levels; gray and purple represent ANA positivity and negativity, respectively. **(B)** ANA positivity rate at different TPOAb levels. **(C)** Correlation between TPOAb levels and ANA positivity risk. ANA, anti-nuclear antibody; TPOAb, thyroid peroxidase antibodies; CI: confidence interval; TSH: thyroid-stimulating hormone. ^a^ P=0.000596, TPOAb level 2 compared with TPOAb level 1; ^b^ P=3.27×10^-11^, TPOAb level 3 compared with TPOAb level 1; ^c^ P=0.002459, TPOAb level 3 compared with TPOAb level 2.

There were 1,659 (12.48%), 82 (17.90%), and 55 (28.50%) patients with positive ANA in TPOAb levels 1, 2, and 3, respectively. The ANA positivity rate significantly varied among different TPOAb levels (χ^2^ = 54.170, P=1.73×10^-12^), and there was a correlation between TPOAb levels and the ANA positivity rate (Kendall’s tau-b=-0.054, P=1.75×10^-7^). Regression analysis demonstrated a significant positive correlation between TPOAb levels and the risk of ANA positivity. The ANA positivity risk at TPOAb level 2 was 53.0% higher than that at TPOAb level 1 (OR=1.530; 95% CI=1.198–1.953). Similarly, the ANA positivity risk at TPOAb level 3 was 179.5% higher than that at TPOAb level 1 (OR=2.795; 95% CI=2.036–3.837). There were 11,636 (87.52%), 376 (82.10%), and 138 (71.50%) patients with negative ANA in TPOAb levels 1, 2, and 3, respectively (**Table 1**; **Figure 2**).

**Table 1:** Comparison of the ANA positivity rate at different TPOAb levels.

TPOAb (IU/mL)	ANA positivity	ANA negativity	Chi-squared test	Kendall’s tau-b
Level 1 (< 150) (*n*=13295)	1659 (12.48%)	11636 (87.52%)	χ*^2^ =* 54.170 *P*=1.73×10^-12^	Kendall’s tau-b=-0.054 *P=*1.75×10^-7^
Level 2 (150–600) (*n*=458)	82 (17.90%)	376 (82.10%)		
Level 3 (> 600) (*n*=193)	55 (28.50%)	138 (71.50%)		
Level 1 vs level 2	χ^2^ = 11.788; OR=0.654; 95%CI=0.512-0.835; *P*=0.000596			
Level 1 vs level 3	χ^2^ = 44.008; OR=0.358; 95%CI=0.261-0.491; *P*=3.27×10^-11^			
Level 2 vs level 3	χ^2^ = 9.171; OR=0.547; 95% CI= 0.369–0.811; *P*=0.002459			

TPOAb, thyroid peroxidase antibodies; ANA, anti-nuclear antibody.

### TPOAb and IgG

Data on TPOAb and IgG were obtained for 13,852 patients (12,502 from the NHANES database), with 309/13,852 (2.23%) patients having IgG levels ≥ 15 g/L.

**Figure 3:**
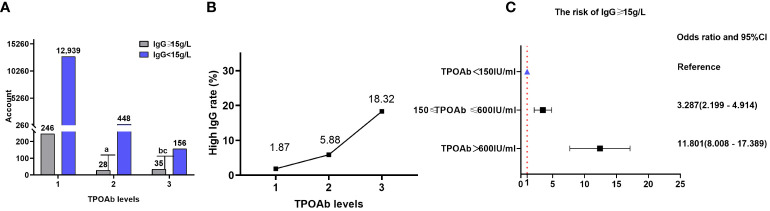
IgG and TPOAb levels. **(A)** Comparison of the proportions of patients with high (≥ 15 g/L) and low (<15 g/L) IgG levels at different TPOAb levels; gray and purple indicate high and low IgG levels, respectively. **(B)** Proportion of patients with high IgG levels at different TPOAb levels. **(C)** Correlation between TPOAb levels and the risk of having IgG levels. TPOAb, thyroid peroxidase; IgG, immunoglobulin G; CI, confidence interval. ^a^ P=8.21×10^-10^, TPOAb level 2 compared with TPOAb level 1; ^b^ P=3.10×10^-23^, TPOAb level 3 compared with TPOAb level 1; ^c^ P=6.8×10^-7^, TPOAb level 3 compared with TPOAb level 2.

There were 246 (1.87%), 28 (5.88%), and 35 (18.32%) patients with high IgG (IgG≥15g/L) in TPOAb levels 1, 2, and 3, respectively. The proportion of patients with IgG levels≥15 g/L significantly varied among different TPOAb levels (P=1.618×10^-27^), and TPOAb levels exhibited a correlation with a high proportion of patients with IgG levels ≥15 g/L (Kendall’s tau-b=-0.111, P=1.32×10^-24^). Logistic regression analysis indicated a significant positive correlation between different TPOAb levels and the risk of elevated IgG levels (≥15 g/L). The risk of having elevated IgG levels (≥ 15 g/L) at TPOAb level 2 was 228.7% higher than that at TPOAb level 1(OR=3.287; 95% CI=2.199–4.914). Additionally, the risk of having elevated IgG level (≥ 15 g/L) at TPOAb level 3 increased by 1080.1% compared with that at TPOAb level 1 (OR=11.801; 95% CI=8.008–17.389). There were 12,939(98.13%), 448 (94.12%), and 156 (81.68%) patients with low IgG (<15 g/L) in TPOAb levels 1, 2, and 3, respectively (**Table 2**; **Figure 3**).

**Table 2:** Comparison of the proportion of patients with high (≥ 15 g/L) and low (< 15 g/L) IgG levels in different TPOAb levels.

TPOAb (IU/mL)	IgG ≥ 15 g/L	IgG <15 g/L	Fisher’s exact test	Kendall’s tau-b
Level 1 (< 150) (*n*=13185)	246 (1.87%)	12939 (98.13%)	*P*=1.618×10^-27^	Kendall’s tau-b=-0.111 *P*=1.32×10^-24^
Level 2(150–600) (*n*=476)	28 (5.88%)	448 (94.12%)		
Level 3 (> 600) (*n*=191)	35 (18.32%)	156 (81.68%)		
Level 1 vs level 2	χ^2^ = 37.710; OR=0.304; 95%CI=0.203-0.455; *P*=8.21×10^-10^			
Level 1 vs level 3	OR=0.085; 95%CI=0.058-0.125; *P*=3.10×10^-23^			
Level 2 vs level 3	χ^2^ = 24.671; OR=0.279; 95% CI=0.164–0.473; *P*=6.8×10^-7^			

TPOAb, thyroid peroxidase antibodies; IgG, immunoglobulin G.

### TRAb and ANA

Data on TRAb and ANA were obtained for 689 patients (none from the NHANES database), resulting in an ANA positivity rate of 35.99% (248/689).

**Figure 4:**
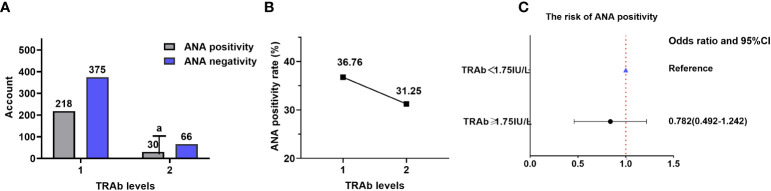
ANA and TRAb levels. **(A)** Comparison of the ANA positivity and negativity rates at different TRAb levels; gray and purple represent ANA positivity and negativity, respectively. **(B)** ANA positivity rate at different TRAb levels. **(C)** Correlation between TRAb levels and ANA positivity risk. TRAb, thyroid-stimulating hormone receptor antibodies; ANA, anti-nuclear antibody; CI, confidence interval; OR, odds ratio. ^a^ P=0.297, TRAb level 2 compared with TRAb level 1.

There were 218 (36.76%) and 30 (31.25%) patients with positive ANA in TRAb levels 1 and 2, respectively. The ANA positivity rate increased as TRAb levels decreased. However, there was no statistically significant difference in the ANA positivity rate among different TRAb levels (χ^2^ = 1.090; OR=1.279; 95% CI=0.805–2.03; P=0.297). Furthermore, there was no correlation between TRAb levels and ANA positivity rate (Kendall’s tau-b=0.040, P=0.284). There were 375 (63.24%) and 66 (68.75%) patients with negative ANA in TRAb levels 1 and 2, respectively (**Table 3**; **Figure 4**).

**Table 3:** Comparison of the ANA positivity rate at different TRAb levels.

TRAb (IU/L)	ANA positivity	ANA negativity	*χ^2^*	*P*	*OR*	95% CI	
<1.75 (*n*=593)	218 (36.76%)	375 (63.24%)	1.090	0.297	1.279	Lower	Upper
≥ 1.75 (*n*=96)	30 (31.25%)	66 (68.75%)				0.805	2.031

TRAb, thyroid-stimulating hormone receptor antibodies; ANA, anti-nuclear antibody; OR, odds ratio; CI, confidence interval.

### TRAb and IgG

Data on TRAb and IgG were obtained for 733 patients (none from the NHANES database), resulting in an IgG level ≥15 g/L in 139/733 (18.96%) patients.

**Figure 5:**
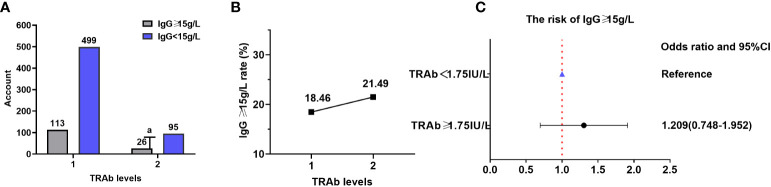
IgG and TRAb levels. **(A)** Comparison of the proportions of patients with high (≥ 15 g/L) and low (< 15 g/L) IgG levels at different TRAb levels; gray and purple represent high and low IgG levels, respectively. **(B)** Proportion of patients with high IgG levels at different TRAb levels. **(C)** Correlation between TRAb levels and the risk of having IgG levels. TRAb, thyroid-stimulating hormone receptor antibodies; IgG, immunoglobulin G; CI, confidence interval; OR, odds ratio. ^a^ P=0.438, TRAb level 2 compared with TRAb level 1.

There were 113 (18.46%) and 26 (21.49%) patients with high IgG (≥15 g/L) in TRAb levels 1 and 2, respectively. The proportion of patients with IgG levels ≥15 g/L did not significantly differ across various TRAb levels (χ^2^ = 0.601, OR=0.827, 95% CI=0.512–1.336, P=0.438). Additionally, there was no correlation between TRAb levels and the proportion of patients with IgG levels ≥15 g/L (Kendall’s tau-b=-0.029, P=0.456). There were 499 (81.54%) and 95 (78.51%) patients with low IgG (<15 g/L) in TRAb levels 1 and 2, respectively (**Table 4**; **Figure 5**).

**Table 4:** Comparison of the proportion of patients with IgG ≥ 15 g/L in different TRAb levels.

TRAb (IU/L)	IgG ≥ 15 g/L	IgG < 15 g/L	*χ^2^*	*P*	OR	95% CI	
<1.75 (*n*=612)	113 (18.46%)	499 (81.54%)	0.601	0.438	0.827	Lower	Upper
≥ 1.75 (*n*=121)	26 (21.49%)	95 (78.51%)				0.512	1.336

TRAb, thyroid-stimulating hormone receptor antibodies; IgG, immunoglobulin G; OR, odds ratio; CI, confidence interval.

### TSH and ANA

Data on TSH and ANA were obtained for 19,426 patients (14,986 from the NHANES database), resulting in an ANA positivity rate of 21.22% (4,123/19,426).

**Figure 6:**
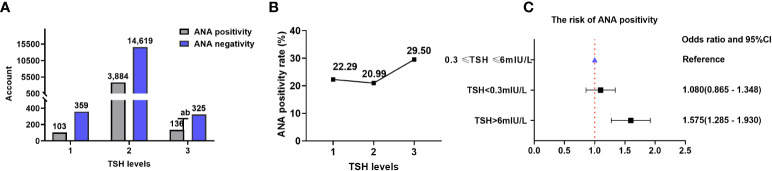
ANA and TSH levels. **(A)** Comparison of the ANA positivity and negativity rates at different TSH levels; gray and purple indicate ANA positivity and negativity, respectively. **(B)** ANA positivity rate at different TSH levels. **(C)** Correlation between TSH levels and ANA positivity risk. TSH, thyroid-stimulating hormone; ANA, anti-nuclear antibody; CI, confidence interval. ^a^ P=0.012, TSH level 3 compared with TSH level 1; ^b^ P=0.00001, TSH level 3 compared with TSH level 2.

There were 103 (22.29%), 3,884 (20.99%), and 136 (29.50%) patients with positive ANA in TSH levels 1, 2, and 3, respectively. The ANA positivity rate significantly varied among different TSH levels (χ^2^ = 19.806, P=0.00005), with a positive correlation between TSH levels and ANA positivity rate (Kendall’s tau-b=-0.019, P=0.013). Logistic regression analysis revealed that high TSH levels influenced the ANA positivity rate. The ANA positivity risk at TSH level 3 increased by 57.5% compared with that at TSH level 2 (OR=1.575, 95%CI=1.285-1.930). There were 359 (77.71%), 14,619 (79.01%), and 325 (70.50%) patients with negative ANA in TSH levels 1, 2, and 3, respectively (**Table 5**; **Figure 6**).

**Table 5:** Comparison of the ANA positivity at different TSH levels.

TSH (mIU/L)	ANA positivity	ANA negativity	Chi-square test	Kendall’s tau-b
Level 1 (< 0.3) (*n*=462)	103 (22.29%)	359 (77.71%)	χ^2^ = 19.806 *P*=0.00005	Kendall’s tau-b=-0.019 *P*=0.013
Level 2 (0.3–6) (*n*=18503)	3884 (20.99%)	14619 (79.01%)		
Level 3 (> 6) (*n*=461)	136 (29.50%)	325 (70.50%)		
Level 1 vs level 3	χ^2^ = 6.245; OR=0.686; 95% CI=0.510–0.922; *P*=0.012			
Level 2 vs level 3	χ^2^ = 19.500; OR=0.635; 95% CI=0.518–0.778; *P*=0.00001			

TSH, thyroid-stimulating hormone; ANA, anti-nuclear antibody.

### TSH and IgG

Data on TSH and IgG were obtained for 19,029 patients (14,986 from the NHANES database), resulting in IgG levels ≥15 g/L in 772/19,029 (4.06%) patients.

**Figure 7:**
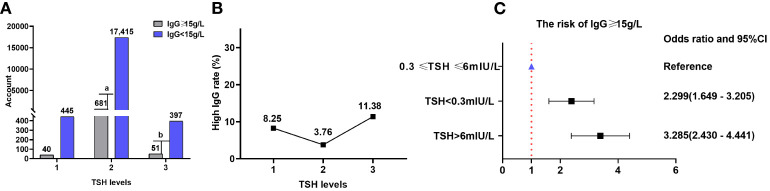
IgG and TSH levels. **(A)** Comparison of the proportions of patients with high (≥ 15 g/L) and low (< 15 g/L) IgG levels at different TSH levels; gray and purple represent high and low IgG levels, respectively. **(B)** Proportion of patients with high IgG levels at different TSH levels. **(C)** Correlation between TSH levels and the risk of having high IgG levels. TSH, thyroid-stimulating hormone; IgG, immunoglobulin G; CI, confidence interval. ^a^ P=4.51×10^-7^, TSH level 2 compared with TSH level 1; ^b^ P=2.77×10^-16^, TSH level 3 compared with TSH level 2.

There were 40 (8.25%), 681 (3.76%), and 51 (11.38%) patients with high IgG (≥15 g/L) in TSH levels 1, 2, and 3, respectively. The proportion of patients with IgG levels ≥15 g/L increased with TSH levels. The proportion of patients with IgG levels ≥15 g/L was significantly different at various TSH levels (χ^2^ = 87.680, P=9.13×10^-20^). However, TSH levels did not correlate with the proportion of patients with IgG levels ≥15 g/L (Kendall’s tau-b=-0.015, P=0.178). The risks of having IgG levels ≥15 g/L at TSH levels 1 and 3 increased by 129.9% (OR=2.299; 95% CI=1.649–3.205) and 228.5% (OR=3.285; 95% CI=2.430–4.441), respectively, compared with that at TSH level 2. There were 445 (91.75%), 17,415 (96.24%), and 397 (88.62%) patients with low IgG (< 15 g/L) in TSH levels 1, 2, and 3, respectively (**Table 6**; **Figure 7**).

**Table 6:** Comparison of the proportion of patients with high (≥ 15 g/L) and low (< 15 g/L) IgG levels at different TSH levels.

TSH (mIU/L)	IgG ≥ 15 g/L	IgG <15 g/L	Chi-squared test	Kendall’s tau-b
Level 1 (< 0.3) (*n*=485)	40 (8.25%)	445 (91.75%)	χ*^2^ =* 87.680 *P*=9.13×10^-20^	Kendall’s tau-b=-0.015 *P*=0.178
Level 2 (0.3–6) (*n*=18096)	681 (3.76%)	17415 (96.24%)		
Level 3 (> 6) (*n*=448)	51 (11.38%)	397 (88.62%)		
Level 1 vs level 2	χ^2^ = 25.465; OR=2.299; 95% CI=1.649–3.205; *P*=4.51×10^-7^			
Level 2 vs level 3	χ^2^ = 66.962; OR=0.304; 95% CI=0.225–0.411; *P*=2.77×10^-16^			

TSH, thyroid-stimulating hormone; IgG, immunoglobulin G.

## Discussion

Thyroid antibodies associated with GD and HT (clinically common AITDs) include TRAb (18) and TPOAb (6), respectively. TPOAb, TRAb, and ANAs are primarily consist of IgG antibodies (19). ANAs are a spectrum of autoantibodies that react with various nuclear molecules, including deoxyribonucleic acid (DNA), ribonucleic acid, histones, acidic nuclear proteins or complexes of these molecular elements (20). A group of data from European population showed that the prevalence of ANA positivity was significantly higher in patients with AITD compared with the control group (45% vs. 14%) (21). Another author reported the ANA positivity rate was only 9% in healthy persons (22). The data from community population in Australia indicated that, in persons from 55 to 85 years, TPOAb positivity rate was 8.4% and ANA positivity rate was 27.9% (23). A study conducted by Pedro et al. from Brazil reported that positive correlation was noted between anti-dsDNA with TPOAb (24). Up to now, the literatures involving the relation between TPOAb and ANA have not been found. Hence, the clinical significance of ANAs in HT remains unknown.

In this paper, we investigated the ANA positivity rate in various TPOAb levels. The results showed that TPOAb levels impacted the percentage of ANA positivity. ANA positivity rate was 12.48% when TPOAb<150IU/mL, 17.90% when 150IU/mL≤TPOAb ≤ 600IU/mL, 28.50% when TPOAb>600IU/mL. For the first time, the outcome showed ANA positivity rate increased with an elevated TPOAb levels. In contrast to other research, we found ANA positivity rate was relatively low in our TPOAb positive patients, which reason was unclear.

An Italy retrospective investigation was conducted the ANA positivity rate arrived 80% in 265 patients with GD (25). Another Italy research assessed the prevalence of ANA in 96 pediatric patients with AITD, in which 86 children with chronic lymphocytic thyroiditis and 7 with GD, the ANA positivity was 71% (26). But, in a Brazil prospective research, 154 patients with AITD (70 HT and 84 GD) were followed for the mean period of 5 years, positive ANA was found in 17.5% (27/154) (27).

It has not been found that literatures reported comparing the ANA positivity rates between TRAb-positive and TRAb-negative individuals in general patients. But some study results from Bulgaria and Iran indicated that, compared patients with GD with healthy persons, the percentage of ANA positivity did not show any significant difference (28, 29).

In current large sample study, when TRAb<1.75IU/L, ANA positivity rate was 36.76%. When TRAb≥1.75IU/L, ANA positivity rate was 31.25%. There was no correlation observed between TRAb levels and ANA positivity rate in our study, which was similar with the data from Bulgaria and Iran(28.29). In our study, the ANA positivity rate in patients with elevated TRAb levels was roughly higher than that reported in Brazil (27) but lower than that reported in Italy (26).

Based on the above analysis, we could conclude that the correlation was significant between TPOAb levels and ANA positivity rate, whereas TRAb levels did not exhibit the same consistency. This suggests the pathophysiological mechanisms underlying different AITDs are not completely uniform.

Additionally, we found higher ANA positivity rates in the range of TSH > 6 mIU/L than in the range of 0.3 mIU/L ≤ TSH ≤ 6 mIU/L. But for ANA positivity rates, there were no significant difference between TSH<0.3mIU/L and 0.3 mIU/L ≤ TSH ≤ 6 mIU/L. It can be inferred from this that ANA positivity is more likely to occur in patients with elevated TSH levels, such as those with hypothyroidism. Clinically, HT usually evolves into a primary hypothyroidism (30), which causes elevated TSH levels. Torok et al. reported that approximately 14% of ANA-positive children without rheumatologic disease exhibit hypothyroidism (31). The reports have not been found involving exploration of the relation between primary hypothyroidism and ANA positivity rate. In all, the results between TSH levels and ANA positivity rate supports the above outcome about ANA positivity rate related to various TPOAb levels, but not TRAb levels.

The current study demonstrated a correlation between TPOAb levels and ANA positivity and IgG levels, with the ANA positivity rate increasing as TPOAb levels increased. The elevated TPOAb levels indicated thyroid follicular epithelial cell injury, suggesting that ANA positivity may be related to thyroid follicular epithelial cell injury. Furthermore, TPOAb levels were significantly associated with ANA positivity risk and high IgG levels. Therefore, since TPOAb serves as a biomarker for HT, ANA positivity may be intrinsically related to and involved in HT’s pathological processes, but not that of GD.

Researches based genetic material have become important evidences for elucidating the pathophysiological mechanisms of diseases. Found by studies that the SNPs rs3177928 and rs7197 were correlated with AITD and GD (compared with the healthy control group), but not with HT (32). The association of the SNP CD40 C/T-1 was evaluated in patients with AITD, and a significant association for CD40 C/T-1 polymorphism with GD was found, but not with HT. Individuals carried the C/C or C/T genotype had a significantly higher risk of GD than those with the T/T genotype, while no association was observed in HT (33). It was pointed out by Frid from Memorial University of Newfoundland, of all the HLA subtypes, HLA-DR3 was the most strongly associated with AITD, especially since 40–50% of patients with GD harbor the HLA-DR3 gene, in contrast to 15–30% of the general population (34). But data on HLA haplotypes in HT have been less definitive than in GD (35). The findings of these studies support our research conclusions from the perspective of genetic materials, which exhibited the pathophysiological mechanisms underlying GD and HT are inconsistent.

This study had some limitations. It was a retrospective study that included patient data spanning a considerable time period from the hospital database, making data normalization challenging. Therefore, future, prospective studies are needed to address this limitation. Additionally, our findings indicate a correlation only between HT and ANA, not between GD and ANA. However, this correlation and its underlying mechanism require further validation.

In summary, based on our research and analysis, the conclusion can be demonstrated that although both HT and GD belong to AITD, their pathophysiological mechanisms are different or distinct. Therefore, when constructing intervention strategies or developing guideline for clinical practice, it is necessary to approach HT and GD in a more targeted and differentiated manner. More importantly, it is not advisable to treat HT and GD as a single issue and collectively refer to them as AITD in subsequent deeper research. Instead, they should be studied individually to gain a more accurate understanding of their respective pathophysiological mechanisms.

## Data availability statement

The original contributions presented in the study are included in the article/supplementary material. Further inquiries can be directed to the corresponding author.

## Ethics statement

The studies involving humans were approved by The Ethics Review Committee of the People’s Hospital Affiliated to Fujian University of Traditional Chinese Medicine. The studies were conducted in accordance with the local legislation and institutional requirements. Written informed consent for participation was not required from the participants or the participants’ legal guardians/next of kin because the study did not impede the personal privacy of patients and business interests. The requirement for informed consent was waived due to the retrospective design of the study.

## Author contributions

YR: Data curation, Visualization, Writing – original draft. XH: Conceptualization, Methodology, Supervision, Writing – review & editing. LY: Methodology, Writing – review & editing. WH: Formal analysis, Data curation, Investigation, Writing – review & editing. LL: Writing – review & editing. ZW: Writing – review & editing. SH: Writing – review & editing. QC: Writing – review & editing. ZH: Writing – review & editing.
